# Expression of matrix metalloproteinases (MMPs)−2/-7/-9/-14 and tissue inhibitors of MMPs (TIMPs)−1/-2 in bovine cutaneous fibropapillomas associated with BPV-2 infection

**DOI:** 10.3389/fvets.2022.1063580

**Published:** 2022-11-28

**Authors:** Florentina Daraban Bocaneti, Gennaro Altamura, Annunziata Corteggio, Oana Irina Tanase, Mihaela Anca Dascalu, Sorin Aurelian Pasca, Ozana Hritcu, Mihai Mares, Giuseppe Borzacchiello

**Affiliations:** ^1^Department of Public Health, Faculty of Veterinary Medicine, Iasi University of Life Sciences “Ion Ionescu de la Brad”, Iaşi, Romania; ^2^Department of Veterinary Medicine and Animal Productions, University of Naples “Federico II”, Naples, Italy; ^3^Institute of Biochemistry and Cell Biology (IBBC), National Research Council (CNR), Naples, Italy; ^4^Department of Pathology, Faculty of Veterinary Medicine, Iasi University of Life Sciences “Ion Ionescu de la Brad”, Iaşi, Romania

**Keywords:** bovine fibropapilloma, BPV, MMP, TIMP, tumor invasion

## Abstract

**Introduction:**

Bovine papillomaviruses −1/−2 (BPVs) are small non-enveloped double-stranded DNA viruses able to infect the skin of bovids and equids, causing development of neoplastic lesions such as bovine cutaneous fibropapillomas and equine sarcoid. Matrix metalloproteinases (MMPs) are a group of zinc-dependent endopeptidases that degrade basal membrane and extracellular matrix, whose function is essential in physiological processes such as tissue remodeling and wound healing. MMPs activity is finely regulated by a balancing with expression of tissue inhibitors of MMPs (TIMPs), a process that is impaired during tumour development. BPV infection is associated with upregulation of MMPs and /or their unbalancing with TIMPs, contributing to local invasion and impairment of extracellular matrix remodeling in equine sarcoid; however, studies regarding this topic in bovine fibropapillomas are lacking.

**Methods:**

The aim of this study was to perform an immunohistochemical and biochemical analysis on a panel of MMPs and TIMPs in BPV-2 positive bovine cutaneous fibropapillomas vs. normal skin samples.

**Results:**

Immunohistochemistry revealed a cytoplasmic expression of MMP-2 (15/19), a cytoplasmic and perinuclear immunoreactivity of MMP-7 (19/19) and MMP-9 (19/19), along with a cytoplasmic and nuclear pattern of MMP-14 (16/19), accompanied by a cytoplasmic expression of TIMP-1 (14/19) and TIMP-2 (18/19) in tumour samples; western blotting revealed an overexpression of MMP-2 (8/9), MMP-7 (9/9) and MMP-9 (9/9), and a decreased level of MMP-14 (9/9), TIMP-1 (9/9) and TIMP-2 (9/9) in tumour versus normal skin samples. Moreover, gelatine zymography confirmed the expression of pro-active MMP-2 (9/9) and MMP-9 (9/9) and, most importantly, indicated the presence and increased activity of their active forms (82 and 62 kDa, respectively) in tumour samples.

**Discussion:**

This is the first study describing MMPs and TIMPs in bovine cutaneous fibropapillomas and our results suggest that their unbalanced expression in presence of BPV-2 may play a significant role in tumour development. A further analysis of supplementary MMPs and TIMPs could bring new important insights into the papillomavirus induced tumours.

## Introduction

Bovine papillomaviruses−1/-2 (BPV) are small non-enveloped double-stranded DNA viruses that belong to *Papillomaviridae* family, able to infect skin of bovids and equines, causing neoplastic lesions such as bovine cutaneous fibropapillomas and equine sarcoid (1, 2). Interestingly, infiltration and localized invasion are often described in fibroblastic tumors, thus malignant tumors can show substantial invasion and infiltration of lymphatics (3). Although bovine cutaneous fibropapillomas is considered to be self-limiting, in some cases these tumors persist long periods with spreading to other body sites (4).

Various malignancies arise from the areas of inflammation as part of the normal host response, therefore are considered “wounds that do not heal” (5) and associated with impaired activity of different proteins and enzymes, such as matrix metalloproteinases (MMPs). Indeed, in different cancer types has been demonstrated an increased expression or activity of matrix metalloproteinases (MMPs), a family of at least 28 zinc-dependent endopeptidases and their inhibitors known as tissue inhibitors of MMPs (TIMPs), all able to degrade components of extracellular matrix (ECM) proteins such as gelatin, collagen, laminin and fibronectin (6–10). Based on their substrate specificity and cellular localization, the MMPs are classified in collagenases (MMP-1,−4,−8,−13), stromelysins (MMP-3,−10, and−11), gelatinases (MMP-2,−9), membrane type MMPs (MMP-14) and others (8, 10–12). Many of MMPs have been shown to be present in various cancers and close attention has been focused on MMP−2 and−9 since they are overexpressed in a variety of malignant tumors. Thus, in human oncology their expression is often associated with tumor grade and poor patient prognosis (13). Moreover, MMP-2 and MMP-9 are regarded as key enzymes in the degradation of the basement membrane, which consists mainly of type IV collagen (12). MMP-9 (gelatinase) can digest the extracellular matrix, laminin, elastin and vitronectin and is established to be more effective in degrading basement membranes than other MMPs. Interestingly, both MMP-2 and MMP-9 were reported to be overexpressed in equine sarcoid, suggesting that tumor progression could be the result of a mechanism responsible for proliferation and more collagen production (14, 15). In human counterpart, it is documented that the association between Human Papillomavirus (HPV) and MMP-2 and−9 overexpression would be an early event in a multistep process of malignant transformation and could address tumoral progression toward a peculiar molecular pattern (16).

MMP-7 (matrilysin) is one of the smallest MMPs and has been suggested to play a role in early stages of tumorigenesis, since it was detected in many tumors, such as those of the breast and skin (17, 18). Additional evidence for the critical role of MMPs in tumorigenesis has been provided with transgenic and knock-out mice, demonstrating that mice lacking MMP-7 and MMP-2 show reduced tumor progression, while an overexpression of MMP-7 in transgenic mice led to enhanced tumorigenesis in a breast cancer model (18).

MMP-14 or MT1-MMP was originally identified as the extracellular protease responsible for activation of pro-MMP-2 (19). Interestingly, in BPV positive equine sarcoid an up-regulation of MMP-14 was demonstrated in several studies, which can explain the sarcoids' very or negligibly low capability to metastasize (15, 20). Moreover, in human counterpart, Akgül and co-workers (2006) showed that expression of the HPV- 8 caused overexpression of MMP-14, suggesting a role of this MMP in the development of HPV-8 induced cutaneous tumors (9).

The main inhibitors regulating the function of MMPs both inside and outside the cell are considered tissue inhibitors of metalloproteinases (TIMPs), with four identified members: TIMP-1, TIMP-2, TIMP-3 and TIMP-4, respectively. TIMPs play an important role in many biological processes, including cancer. An imbalance between TIMP and MMP activities is considered to result in excessive degradation of matrix components and tumor invasion (21). Interestingly, in recent studies performed on equine sarcoids, TIMP-2, MMP-2 and MMP-14 were overexpressed when compared to normal skin, suggesting that the imbalance between production and degradation of collagen could play an important role in the pathogenesis of the equine sarcoid (15).

Nowadays, attempts to describe the etiomorphology of bovine cutaneous fibropapillomatosis are well-documented, although the exact intracellular mechanism leading to neoplastic transformation, tumor progression and involution are not fully understood. Different intracellular mechanisms were described to support the cutaneous neoplastic transformation, including activation of phosphatidyl inositol-3-kinase (PI3K)/Akt and Ras-mitogen-activated-protein-kinase-Erk (Ras-MAPK-Erk) pathways, an alteration of bcl-2 and p53 expression or an upregulation of cyclooxygenase-2 or connexin 26 (22–25). Comparatively, it has been hypothesized that in genetically predisposed equines, BPV type−1/2 may be responsible for impaired fibroblast proliferation and for changes in dynamics of the extracellular matrix (ECM) and its main components. These changes could induce a modification of the wound healing process and may therefore be an important factor in the pathogenesis of equine sarcoids (15). Since in literature the data regarding the expression of MMPs and TIMPs in naturally induced BPV tumors are lacking, this study primarily aims to demonstrate an immunohistochemical, biochemical and zymographic expression of MMP-2 and MMP-9 enzymes, as well of MMP-7/-14 and their endogenous inhibitors TIMP-1/-2 both in normal skin samples and in samples collected from bovine suffering from fibropapillomatosis. To the best of authors' knowledge, this study is the first documentation of the expression of MMP-2,−7,−9,−14 and TIMP-1/-2 enzymes in healthy and diseased skin samples in bovines.

## Materials and methods

### Samples

Nineteen fibropapilloma samples were obtained from 19 bovines (T = 19), aged between 7 months to 3 years and were clinically identified according to their gross pathology, consisting of a cauliflower appearance. The lesions were located mainly on the head, lateral neck and shoulders, with a diameter ranging from 0.5 to 3 cm. All samples included in this study were obtained from a public slaughterhouse. In the present study, we used the same tumor samples that were employed in the previous work of Bocaneti Daraban et al. (26) and all tumor samples were known to be BPV-2 positive (26). Additionally, four normal skin samples (NS = 4) were obtained from healthy bovines, harvested from auricular conchae inner skin and further tested separately to detect BPV-1/-2 DNA, following the same protocol as mentioned above; all 4 skin samples resulted to be BPV-1/-2 negative. Tissue samples were fixed in formalin for routine histologic diagnosis and immunohistochemical analysis. Moreover, in order to prevent cross-contamination, for each paraffin-embedded tissue sample, 20 μm thick sections were cut with separate microtome blades and stored in sterile tube for molecular biology analysis. Moreover, samples T1 - T9 and NS1-NS4 were appropriately processed and frozen at −80 °C for western blotting and zymography analysis.

### Histopathology

Fibropapilloma samples were 10 % formalin fixed paraffin-embedded for routine histological processing and stained with haematoxylin and eosin for light microscopy evaluation, according to the guideline proposed by Goldschmidt and Goldschmidt (27).

### Immunohistochemistry

Paraffin sections of 19 fibropapillomas and 4 normal skins were dewaxed in xylene, dehydrated in graded alcohols and washed in 0.01 M phosphate-buffered saline (PBS), pH 7.2–7.4. Endogenous peroxidase was blocked with hydrogen peroxide 0.3 % in absolute methanol for 20 min at room temperature (RT). The immunohistochemical (IHC) protocol (streptavidin biotin- peroxidase method - Novolink Polymer Detection System; Leica Biosystems, NewCastle, United Kindom) was described by the authors in a previous study (23). Primary antibodies used in this study are listed in Table 1. The antibodies anti-MM-2, anti-MMP-7, anti-MMP-9 and anti-MMP-14 were applied for 1 h at RT, while the antibodies anti-TIMP-1 and anti-TIMP-2 were applied overnight at 4 °C. The treatment with diaminobenzidine was used to visualize the specific immunoreactivity. The immunolabelling procedure included negative control sections, where primary antibodies were omitted and incubated with PBS instead. Canine mammary carcinoma samples were employed as positive control, since were demonstrated to express these MMPs and TIMPs (28).

**Table 1:** Primary antibodies used for immunohistochemistry and western blotting analysis.

**Antibody**	**Manufacturer**	**Catalog number**	**Species reactivity**	**Host/Isotype**	**IHC dilution**	**WB dilution**
MMP-2	ThermoFisher Scientific	PA5-16504 (RRID:AB_10981559)	Bovine, Human, Mouse, Rat	Rabbit/IgG	1:200	1:500
MMP-7	ThermoFisher Scientific	PA5-28076 (RRID:AB_2545552)	Bovine, Human, Mouse, Rat	Rabbit/IgG	1:100	1:1,000
MMP-9	ThermoFisher Scientific	PA5-27191 (RRID:AB_2544667)	Bovine, Human, Mouse	Rabbit/IgG	1:200	1:1,000
MMP-14	ThermoFisher Scientific	PA5-104459 (RRID:AB_2853760)	Human, Mouse, Rat	Rabbit/IgG	1:200	1:1,000
TIMP-1	Santa Cruz	2A5:sc-21734 (RRID:AB_628359)	Human, Mouse, Rat	Mouse	1:200	1:500
TIMP-2	ThermoFisher Scientific	MA5-12207 (RRID:AB_10979570)	Bovine, Guinea pig, Human, Mouse, Rabbit, Rat	Mouse/IgG2a, kappa	1:200	1:500

MMPs and TIMPs immunoreactivity were scored as previously described by Martano et al. (15): –, negative; +, weak, individual positive cells; ++, foci of moderate positivity; +++, > 50% of cells moderately to strongly positive. The immunoreactivity was scored by two observers (FDB and GB) under blinded conditions.

### Sodium dodecyl sulfate polyacrylamide gel electrophoresis/western blotting

Biochemical analysis was performed on four normal skin samples (NS1–NS4) and nine fibropapilloma samples (T1-T9); tissue biopsies were each cut with separate, disposable scalpel blades and brand-new steel beads were employed for mechanical homogenation. Protein extraction, electrophoresis and blotting were performed as previously described by Altamura et al. (29). The membranes were blocked with 5% bovine serum albumin (BSA) in TBS-0.1% Tween buffer (10 mM Tris–HCl, pH 7.4, 165 mM NaCl, 0.1% Tween) at RT, washed with TBS-0.1% Tween, and incubated with the following primary antibodies: anti-MMP-2 (1:500), anti-MMP-7 (1:1,000), anti-MMP-9 (1:1,000), anti-MMP-14 (1:1,000), anti-TIMP-1 (1:500) and anti-TIMP-2 (1:500). After appropriate washing steps in TBS-0.1% Tween buffer, goat anti-mouse (GE Healthcare #LNA931V/AH) and donkey anti-rabbit (GE Healthcare #LNA934V/AH) secondary antibodies conjugated with horseradish peroxidase were applied for 1 h at RT, and protein bands were visualized by enhanced chemiluminescence (ECL, Bio-Rad) using ChemiDoc gel scanner (Bio-Rad). The blots were stripped and reprobed against mouse anti-β-actin antibody (C-2: sc-8432, Santa Cruz) at 1:500 dilution to ensure comparable amounts of proteins for each sample. Densitometric analysis for protein quantization was achieved by using Image Lab software (Bio-Rad). The protein concentrations were normalized to the β-actin levels and expressed as the densitometric ratio.

### Gelatinase zymography

The enzymatic activity of MMP-2 and MMP-9 was analyzed by gelatin zymography, with 7.5 % polyacrylamide gel containing 0.1% gelatine (Sigma Aldrich) as substrate. Twenty μg of each protein extract sample were diluted 1:1 in non-reducing loading buffer (Bio-Rad) and electrophoresed in non-reducing electrophoresis conditions at 120 volts for 1 h and 30 min. After electrophoresis, two washing steps of 30 min each were performed in zymogram developing buffer (2.5% Triton X-100, 50 mM Tris-HCl, pH 7.5, 5 mM CaCl_2_, 1 μM ZnCl_2_). Further, the gel was incubated with zymogram incubation buffer containing co-factors necessary for the gelatinase reaction (1% Triton X-100, 50 mM Tris-HCl pH 7.5, 5 mM CaCl_2_, 1 μM ZnCl_2_) overnight at 37 °C and then stained with staining buffer (40% methanol, 10 % acetic acid, 50 ml deionized water, 0.5 g Commasie Blue) for 1 h on a rotary shaker at RT. The gel was destained with destaining solution (40 % methanol, 10 % acetic acid, 500 ml deionized water), until the bands were clearly visible and digital images were acquired using ChemiDoc gel scanner (Bio-Rad Laboratories). Densitometric analysis was performed as described above.

### Statistical analysis

For statistical analysis, Student's *t*-test was performed using SPSS 17.0 software (SPSS Inc.), and differences were considered to be statistically significant for ^*^P < 0.05 or ^**^P < 0.01.

## Results

### Histological diagnosis

**Figure 1:**
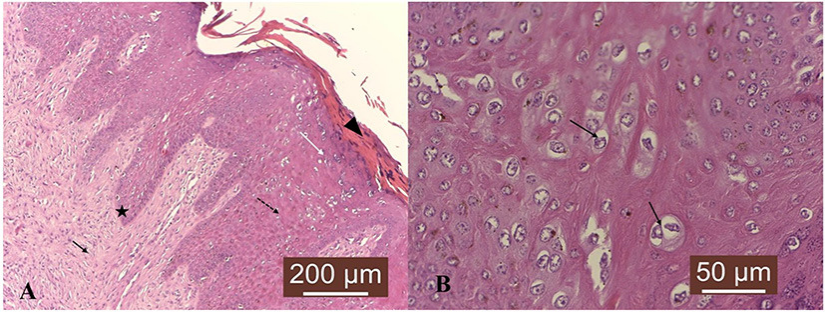
Microscopic characteristics of cutaneous fibropapilloma: **(A)** Mild hyperkeratosis (black arrowhead), epithelial (black dotted arrow and white arrow) and dermal (black arrow) hyperplasia, accompanied by rete pegs (black star); HE, 10 X. **(B)** Scattered koilocytes are found in the spinosum stratum (black arrows). HE, 40 X.

The main lesional characteristics consisted in mild hyperkeratosis, epithelial and dermal hyperplasia, accompanied by rete pegs (Figure 1A). Numerous cells found in the granular epithelial layer displayed keratohyalin granules and cytoplasmic vacuolation was evident in some cells. Moreover, koilocytes were also observed, being suggestive of papillomavirus infection (Figure 1B). Therefore, histological features were consistent with a diagnosis of cutaneous fibropapillomas.

### Immunohistochemistry

The expression patterns of MMP-2, MMP-7, MMP-9, MMP-14, TIMP-1 and TIMP-2 in 19 bovine cutaneous fibropapillomas and 4 normal skin samples are summarized in Table 2.

**Table 2:** Immunoreactivity scoring of MMP-2, MMP-7, MMP-9, MMP-14, TIMP-1, and TIMP-2 in bovine normal skin and fibropapilloma samples.

**Sample**	**BPV – 2 DNA**	**MMP-2**	**MMP-7**	**MMP-9**	**MMP-14**	**TIMP-1**	**TIMP-2**
**NS1**	**-**	**+**	**+**	**+**	**++**	**++**	**++**
**NS2**	**-**	**+**	**+**	**+**	**++**	**++**	**++**
**NS3**	**-**	**+**	**+**	**+**	**++**	**++**	**++**
**NS4**	**-**	**+**	**+**	**+**	**++**	**++**	**++**
**T1**	**+**	**+**	**+**	**++**	**+**	**+**	**+**
**T2**	**+**	**++**	**+**	**++**	**+**	**-**	**+**
**T3**	**+**	**+++**	**++**	**+++**	**+**	**++**	**++**
**T4**	**+**	**+**	**+++**	**+++**	**+**	**-**	**+**
**T5**	**+**	**++**	**+++**	**++**	**+**	**-**	**+**
**T6**	**+**	**+**	**+++**	**+++**	**++**	**+**	**+**
**T7**	**+**	**+++**	**+++**	**++**	**++**	**+**	**++**
**T8**	**+**	**++**	**++**	**+++**	**+**	**+**	**+**
**T9**	**+**	**+**	**+**	**+++**	**+**	**+**	**++**
**T10**	**+**	**-**	**++**	**+++**	**+**	**+**	**+**
**T11**	**+**	**+**	**+**	**++**	**++**	**-**	**+**
**T12**	**+**	**+**	**++**	**+**	**-**	**++**	**++**
**T13**	**+**	**++**	**++**	**+++**	**+**	**+**	**++**
**T14**	**+**	**-**	**+**	**+**	**++**	**-**	**+**
**T15**	**+**	**+++**	**++**	**+++**	**+**	**+**	**++**
**T16**	**+**	**-**	**++**	**++**	**-**	**++**	**+**
**T17**	**+**	**++**	**+++**	**+++**	**+**	**+**	**++**
**T18**	**+**	**-**	**+**	**++**	**-**	**++**	**-**
**T19**	**+**	**++**	**+++**	**+++**	**+++**	**+**	**++**

**Figure 2:**
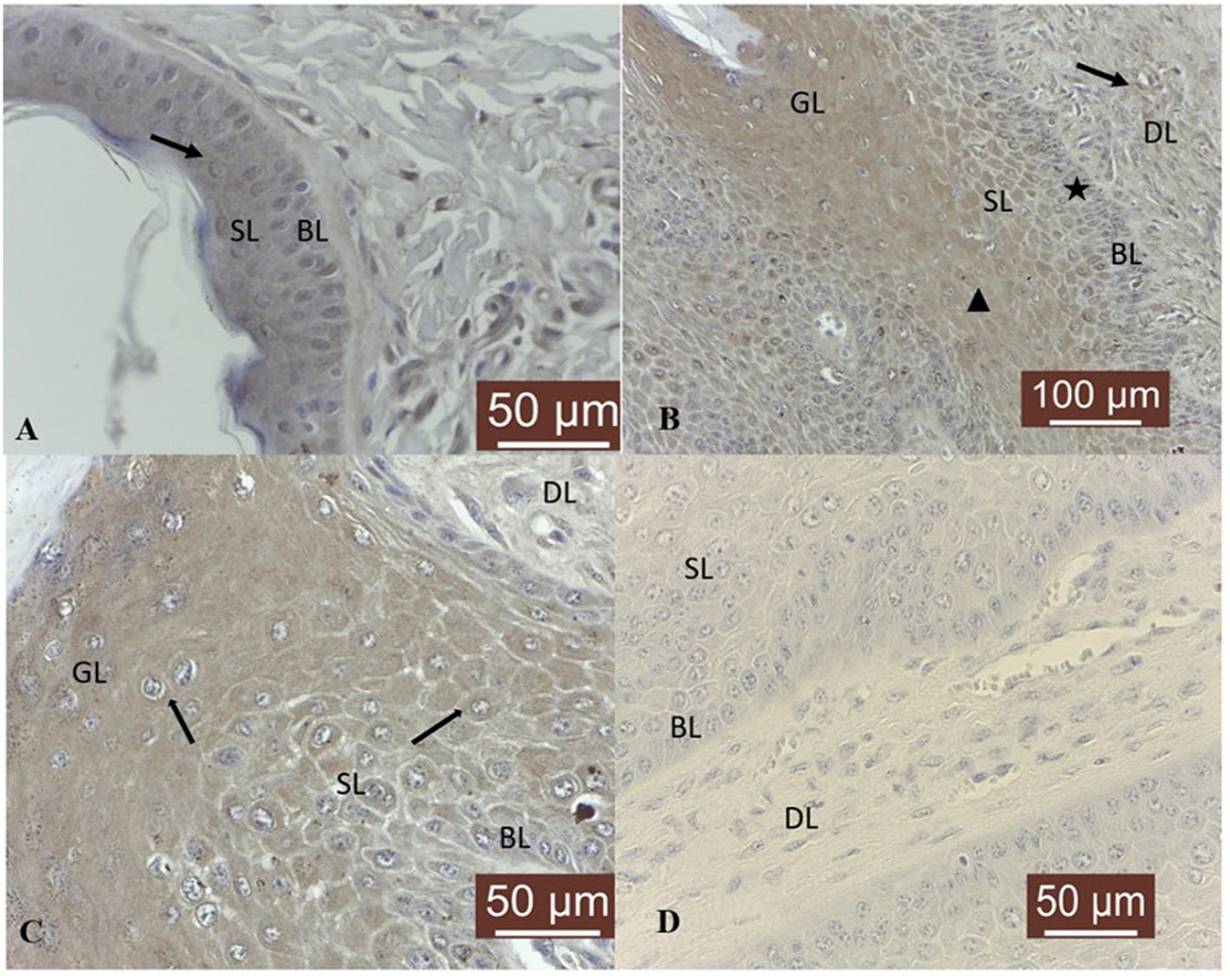
MMP-2 immunostaining in bovine normal skin and fibropapillomas: **(A)** Cytoplasmic and perinuclear expression (black arrow) in normal skin keratinocytes from basal (BL) and spinosum (SL) epithelial layers; 40X. **(B)** Fibropapilloma: MMP-2 is expressed in keratinocytes from basal (BL), spinosum (SL) and granular (GL) epithelial layers (black arrowhead), although a weaker immunoreactivity is noted in the basal cell layer (black star); note a moderate positivity in cytoplasm of SL and GL cells and in fibroblast cells (black arrow) from dermal layer (DL); 20 X. **(C)** MMP-2 is strongly expressed in a fine light brown granular cytoplasmic (black arrows) and perinuclear pattern; 40 X. **(D)** Negative control: fibropapilloma with the first antibody anti-MMP-2 omitted and incubated with secondary antibody did not stain; 40 X.

In 4/4 normal skin samples, MMP-2 was faintly expressed in basal, spinosum and granular epithelial layers, consisting in a fine light brown granular cytoplasmic and perinuclear pattern (Figure 2A). Four out of 19 (21 %) tumor samples did not show any specific immunoreactivity, while in 15/19 (79 %) fibropapillomas, MMP-2 was expressed in the cytoplasm of basal, spinosum and granular epithelial layers, although a weaker immunoreactivity was noted in the basal cell layer (Figure 2B). Fibropapillomas showed a variable immunoreactivity scoring: 6/19 (32 %) samples displayed a weak positivity, 6/19 (32 %) showed a moderate positivity, while 3/19 (16 %) samples displayed a strong immunosignal (Figure 2C). Moreover, in 11/19 (58 %) samples, a moderate labeling was recorded in the dermal layer, in cytoplasm of fibroblast cells (Figure 2B). Fibropapillomas sections with the first antibody anti-MMP-2 omitted and incubated with PBS (negative control) did not exhibit positive staining (Figure 2D).

**Figure 3:**
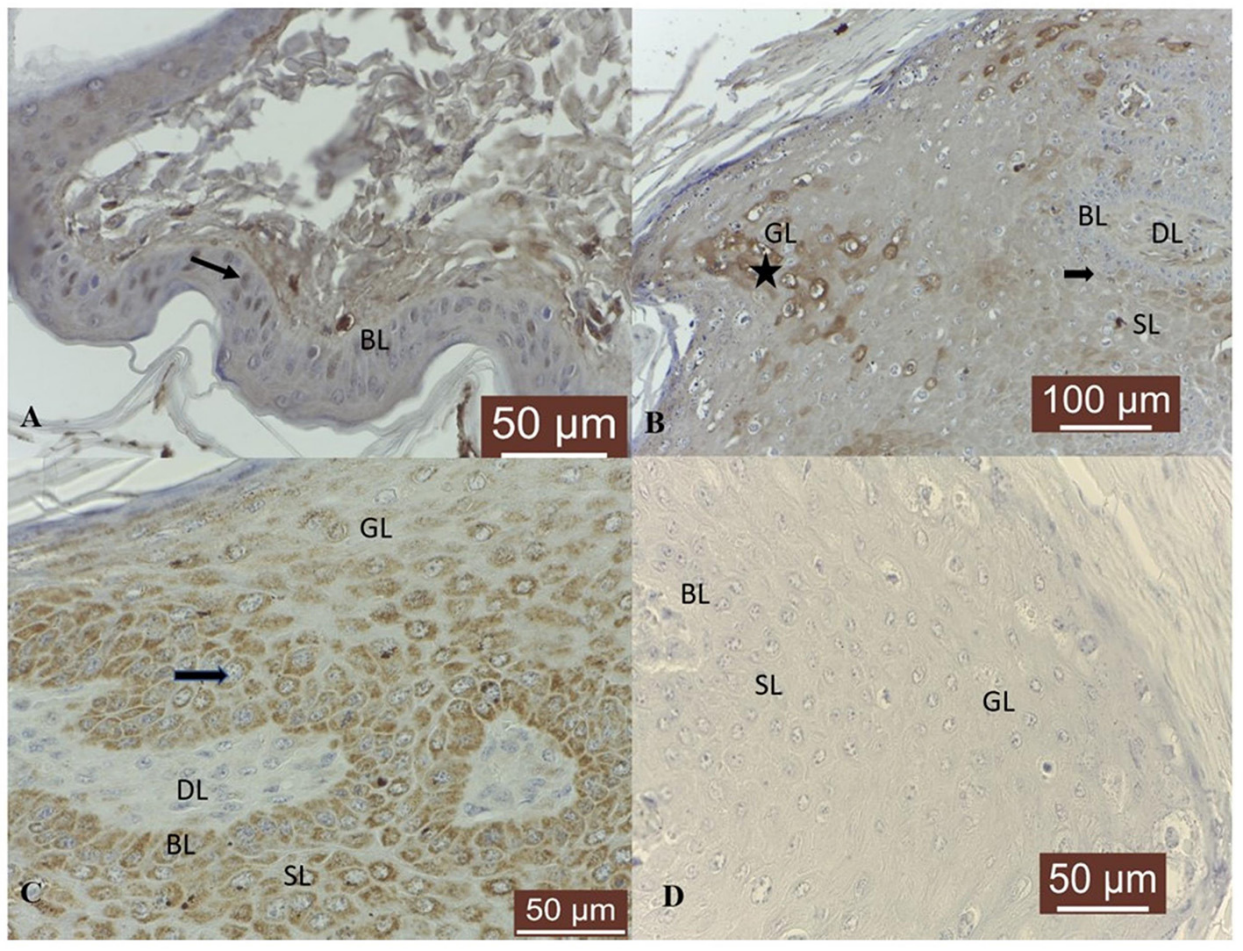
MMP-7 immunostaining in bovine normal skin and fibropapillomas: **(A)** Normal skin: MMP-7 perinuclear and nuclear immunoreactivity (black arrow) predominantly in basal cell layer (BL); 40 X. **(B)** Fibropapillomas: MMP-7 appeared as a finely granular cytoplasmatic (black arrow) and perinuclear staining in keratinocytes from basal (BL), spinosum (SL) and granular (GL) epithelial layers and in 20–30% of fibroblast cells from dermal layer (DL), while scattered keratinocytes from granular layer (GL) showed a strong immunoreactivity (black star); 20 X. **(C)** A detailed MMP-7 strong perinuclear (black arrow) immunosignaling confined to BL, SL and GL epithelial cells; 40 X. **(D)** Negative control: fibropapilloma with the first antibody anti-MMP-7 omitted and incubated with secondary antibody did not stain; 40 X.

Furthermore, MMP-7 was expressed in all normal skin samples, exhibiting predominantly a moderate cytoplasmic, perinuclear and nuclear immunoreactivity in basal and parabasal layers (Figure 3A). Nineteen out of 19 (100 %) fibropapilloma samples showed specific cytoplasmic and perinuclear immunoreactivity, with a variable degree of expression confined to basal, spinosum and granular epithelial layers (Figure 3B); a strong positivity was recorded in 6/19 (32 %) samples (Figure 3C), a moderate intensity was noted in 7/19 samples (36 %), while 6/19 (32 %) of samples showed a weak immunolabeling. Four out of nineteen 4/19 (21 %) tumor samples showed a weak cytoplasm expression in fibroblasts from dermal layer (Figure 3B). However, the nuclear pattern seen in the normal skin samples was not observed in the fibropapillomas. The tumor sections with the first antibody anti-MMP-7 omitted and incubated with PBS (negative control) did not exhibit positive staining (Figure 3D).

**Figure 4:**
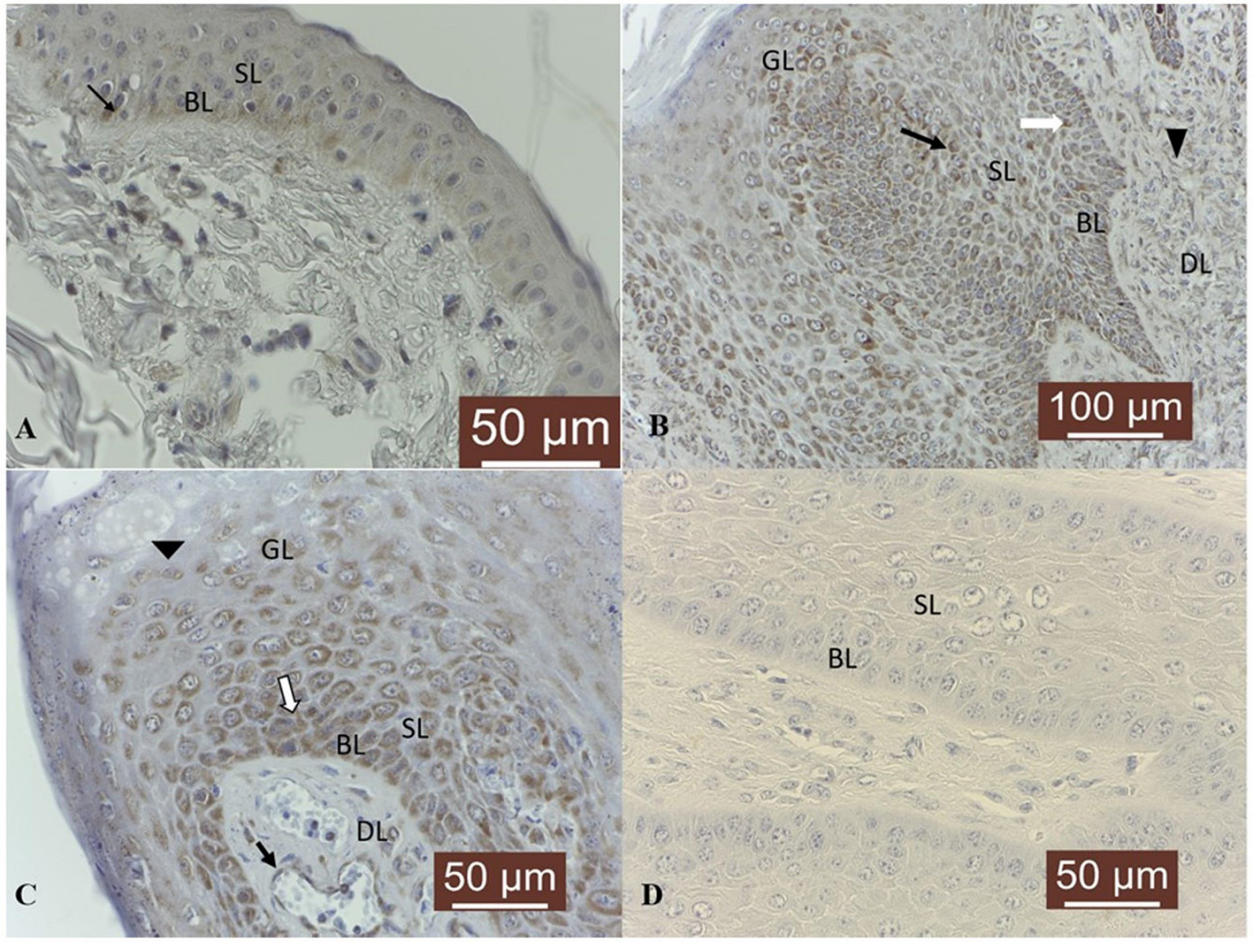
MMP-9 immunostaining in bovine normal skin and fibropapillomas: **(A)** In bovine normal skin, a weak specific immunoreactivity for MMP-9 is noted in the cytoplasm of cells from basal cell layer (BL) (black arrow); 40 X. **(B)** In bovine fibropapillomas, MMP-9 is expressed by keratinocytes from basal (BL) (white arrow), spinosum (SL) (black arrow) and granular (GL) epithelial layers and fibroblast cells (black arrowhead) from dermal layer (DL); 20 X. **(C)** Strong cytoplasmic and perinuclear (white arrow) immunoreactivity of MMP-9 in keratinocytes from BL, SL, and GL (black arrowhead) of fibropapilloma; note the positivity of blood vessels (black arrow); 40 X. **(D)** Negative control: fibropapilloma with the first antibody anti-MMP-9 omitted and incubated with secondary antibody did not stain; 40 X.

MMP-9 was expressed predominantly in the cytoplasm of basal cell layer in 4/4 skin samples, where a weak immunoreactivity was recorded (Figure 4A). In tumor samples, a cytoplasmic and perinuclear immunoreactivity was evident in epithelial cells from basal, spinosum and granular epithelial layers and fibroblast cells from dermal layer (Figure 4B), scoring from a weak expression in 2/19 (11 %) tumors, to moderate in 7/19 (36 %) samples and strong in 10/19 (53 %) samples. Indeed, an intense immunoreactivity was observed in the basal cell layer, concomitantly with scattered cells from spinosum stratum that displayed the same pattern. Moreover, a moderate positivity was noted in the dermal blood vessels (Figure 4C). The negative controls consisting in fibropapillomas sections with the first antibody anti-MMP-9 omitted and incubated with PBS did not exhibit positive staining (Figure 4D).

**Figure 5:**
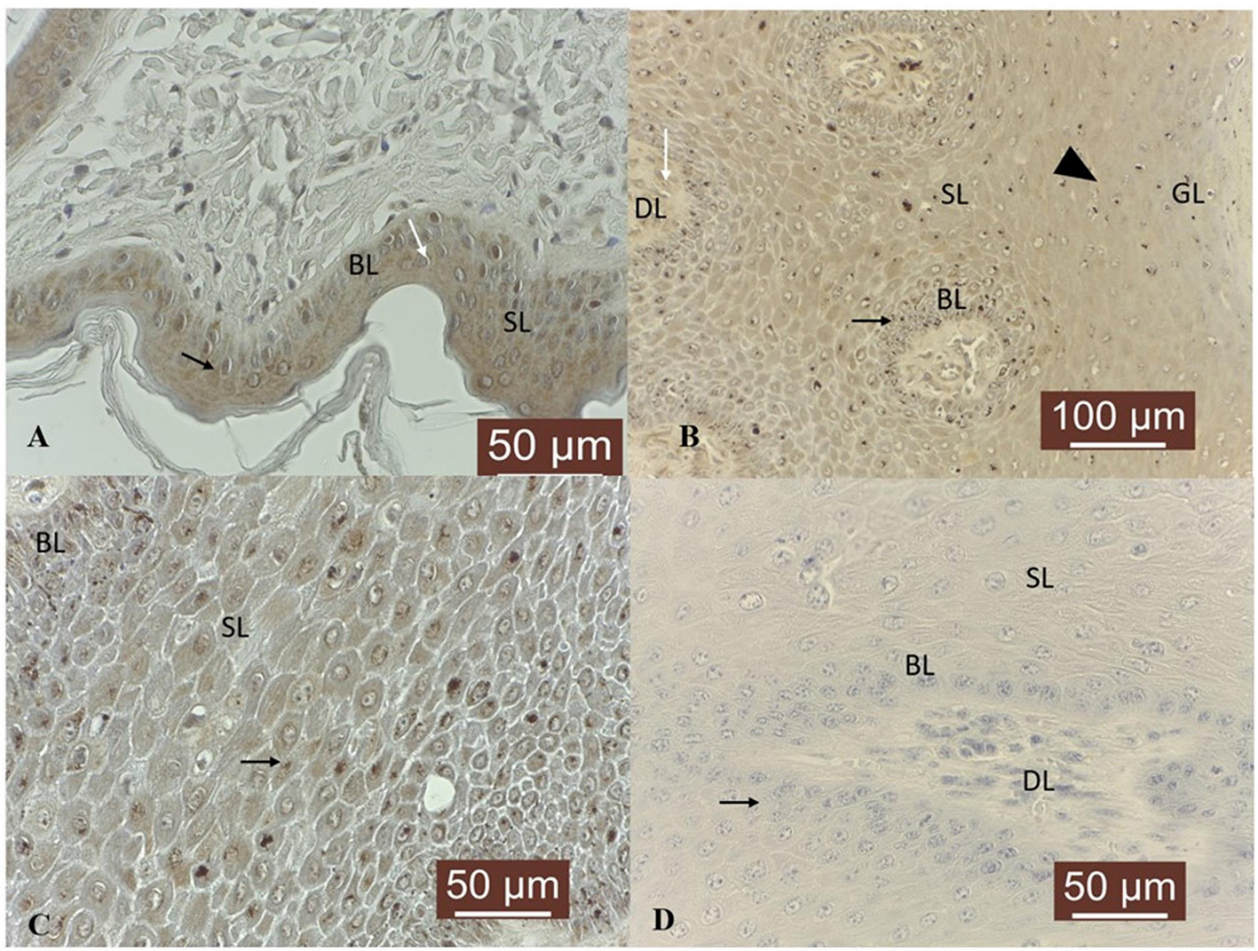
MMP-14 immunostaining in bovine normal skin and fibropapillomas: **(A)** In normal skin samples, MMP-14 showed a specific immunoreactivity in a cytoplasmic, perinuclear (black arrow) and nuclear (white arrow) pattern in basal (BL) and spinosum (SL) epithelial cell; 40 X. **(B)** In fibropapillomas, MMP-14 was moderate expressed in cytoplasm (black arrow) of cells from basal (BL), spinosum (SL) and granular (GL) (black arrowhead) layers, while the fibroblasts from dermal layer (DL) showed a weak positivity (white arrow); 20 X. **(C)** Note a detailed fine granular cytoplasmic, perinuclear (black arrow) and nuclear pattern in tumor keratinocytes from BL and SL; 40 X. **(D)** Negative control: fibropapilloma with the first antibody anti-MMP-14 omitted and incubated with secondary antibody did not stain; 40 X.

With respect to MMP-14, in 4/4 skin samples its expression was detected as moderate cytoplasmic, perinuclear and nuclear immunosignal in basal, spinosum and granular epithelial layers (Figure 5A). Fibropapillomas featured a weak positivity in 11/19 (58 %) samples, moderate in 4/19 (21 %) and strong in 1/19 (5 %) sample (T19), characterized by a finely granular pattern in the cytoplasm (Figure 5B) and interestingly, in the nucleus (Figure 5C) of some keratinocytes cells from spinosum and granular layers. In 5/19 (26 %) fibropapillomas, a weak immunoreactivity was detected in the cytoplasm (Figure 5B) of fibroblast from dermal layer. The tumor sections with the first antibody anti-MMP-14 omitted and incubated with PBS (negative control) did not exhibit positive staining (Figure 5D).

**Figure 6:**
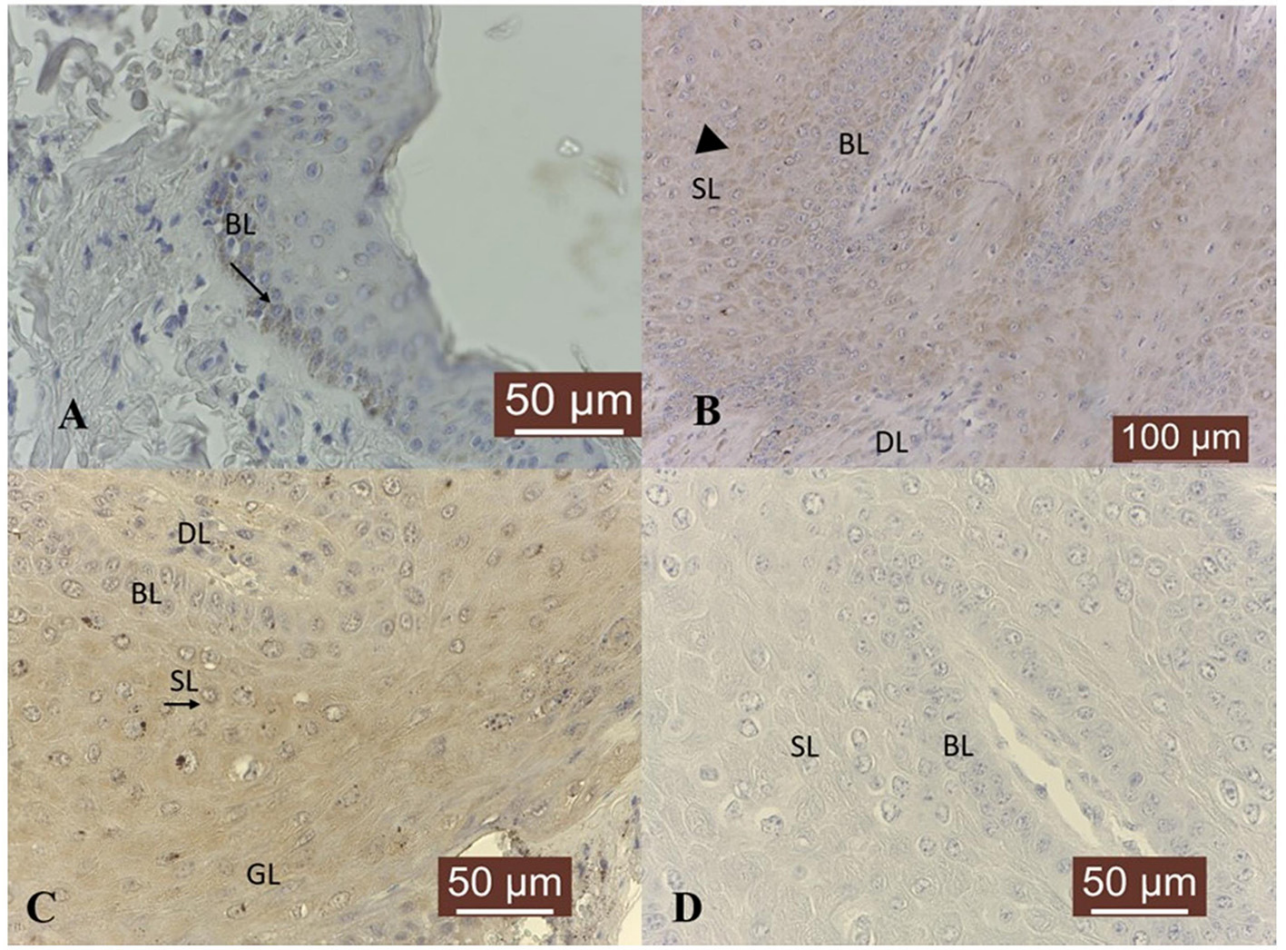
TIMP-1 immunoexpression in bovine normal skin and fibropapillomas: **(A)** TIMP-1 is detected as a moderate cytoplasm (black arrow) pattern in normal skin basal cell layer (BL); 40 X. **(B)** In tumor samples, TIMP-1 expression was recorded as a weak finely granular staining pattern in the cytoplasm of keratinocytes from basal (BL), spinosum (SL) (black arrowhead) and granular (GL) layers, while few fibroblasts from dermal layer (DL) showed a weak immunoreactivity; 20 X. **(C)** Note a moderate granular cytoplasmic pattern (black arrow) in spinosum layer of tumor; 40 X. **(D)** Negative control: fibropapilloma with the first antibody anti-TIMP-1 omitted and incubated with secondary antibody did not stain; 40 X.

In 4/4 normal skin samples, a moderate cytoplasmic TIMP-1 immunoreactivity was noted in epidermis, more precisely in basal cells and only few cells from spinosum and granular layers showed moderate positivity (Figure 6A). In 5/19 (26 %) tumor samples, no specific TIMP-1 immunoreactivity could be detected. However, its expression was variable in the rest of tumor samples: weak in 10/19 (53 %) and moderate in 4/19 (21 %); a finely granular staining pattern was recorded in the cytoplasm of keratinocytes from basal, spinosum and granular layers (Figure 6B), as well as in 20–30 % of fibroblasts from the dermal layer (Figure 6C). The tumor sections with the first antibody anti-TIMP-1 omitted and incubated with PBS (negative control) did not exhibit positive staining (Figure 6D).

**Figure 7:**
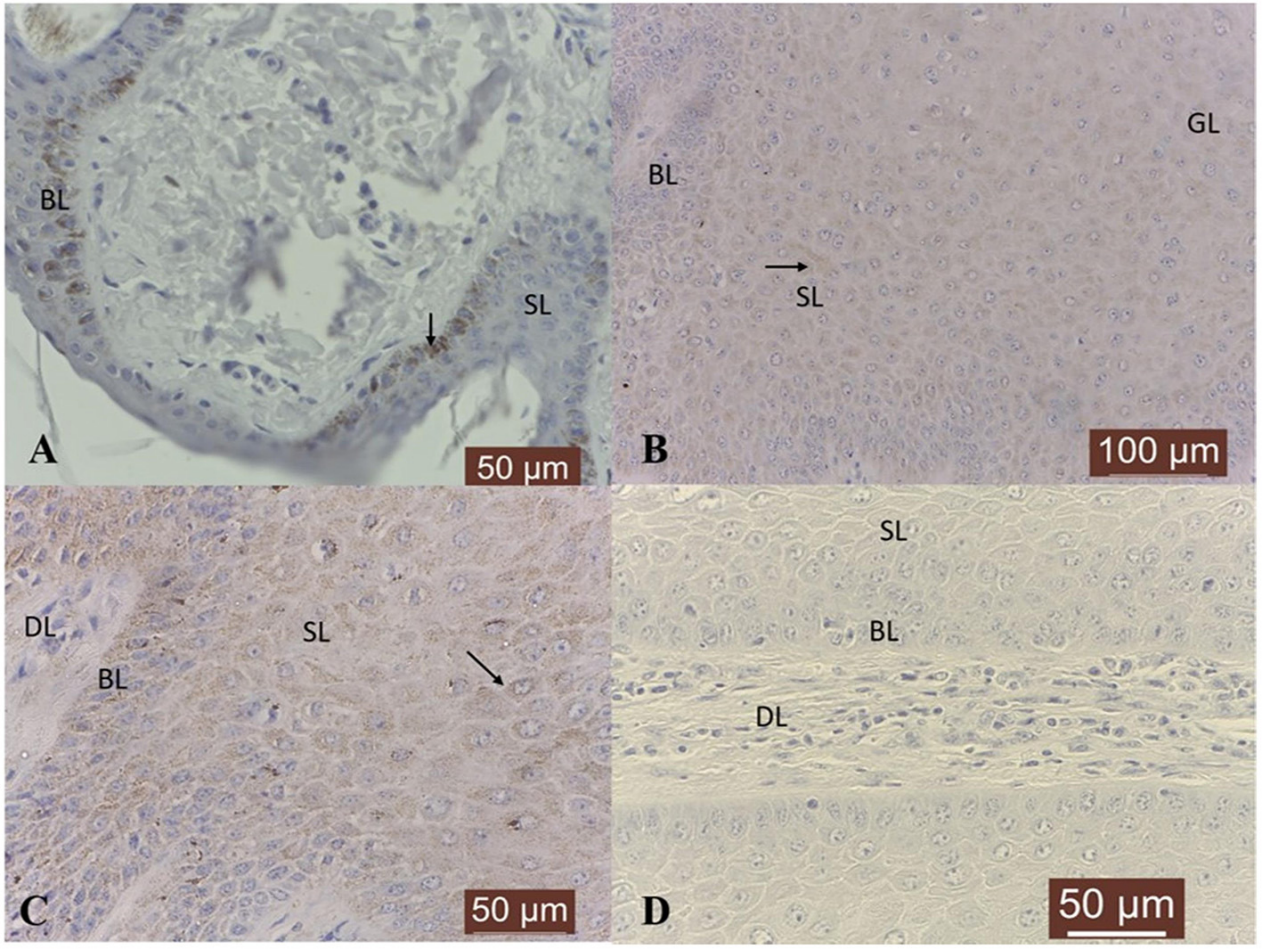
TIMP-2 immunoexpression in bovine normal skin and fibropapillomas: **(A)** TIMP-2 moderate granular cytoplasmic pattern (black arrow) mainly in basal layer (BL) of normal skin; 40 X. **(B)** Most tumor cells from spinosum (SL) and granular (GL) layers exhibited cytoplasmic (black arrow) TIMP-2 immunosignal; 20 X. **(C)** Note a weak to moderate granular cytoplasmic and perinuclear pattern (black arrow) in keratinocytes from BL and SL; 40 X. **(D)** Negative control: fibropapilloma with the first antibody anti-TIMP-2 omitted and incubated with secondary antibody did not stain; 40 X.

With respect to TIMP-2 expression, a cytoplasmic moderate immunoreactivity restricted to the basal epithelial cells was detected in all normal skin samples (Figure 7A). Fibropapillomas exhibited predominantly cytoplasmic TIMP-2 immunoreactivity confined to basal, spinosum and granular cell layers (Figure 7B), with intensity ranging from weak in 10/19 (53 %) samples, to moderate in 8/19 fibropapillomas (42 %); in 1/19 (5 %) (sample no T18) no specific immunoreactivity was detected. Most cells from spinosum and granular layers exhibited perinuclear immunosignal (Figure 7C). A weak expression was noted also in the fibroblast cells of almost all samples. In parallel, negative controls represented by fibropapillomas sections with the first antibody anti-TIMP-2 omitted and incubated with PBS did not exhibit positive staining (Figure 7D).

### Western blot analysis

**Figure 8:**
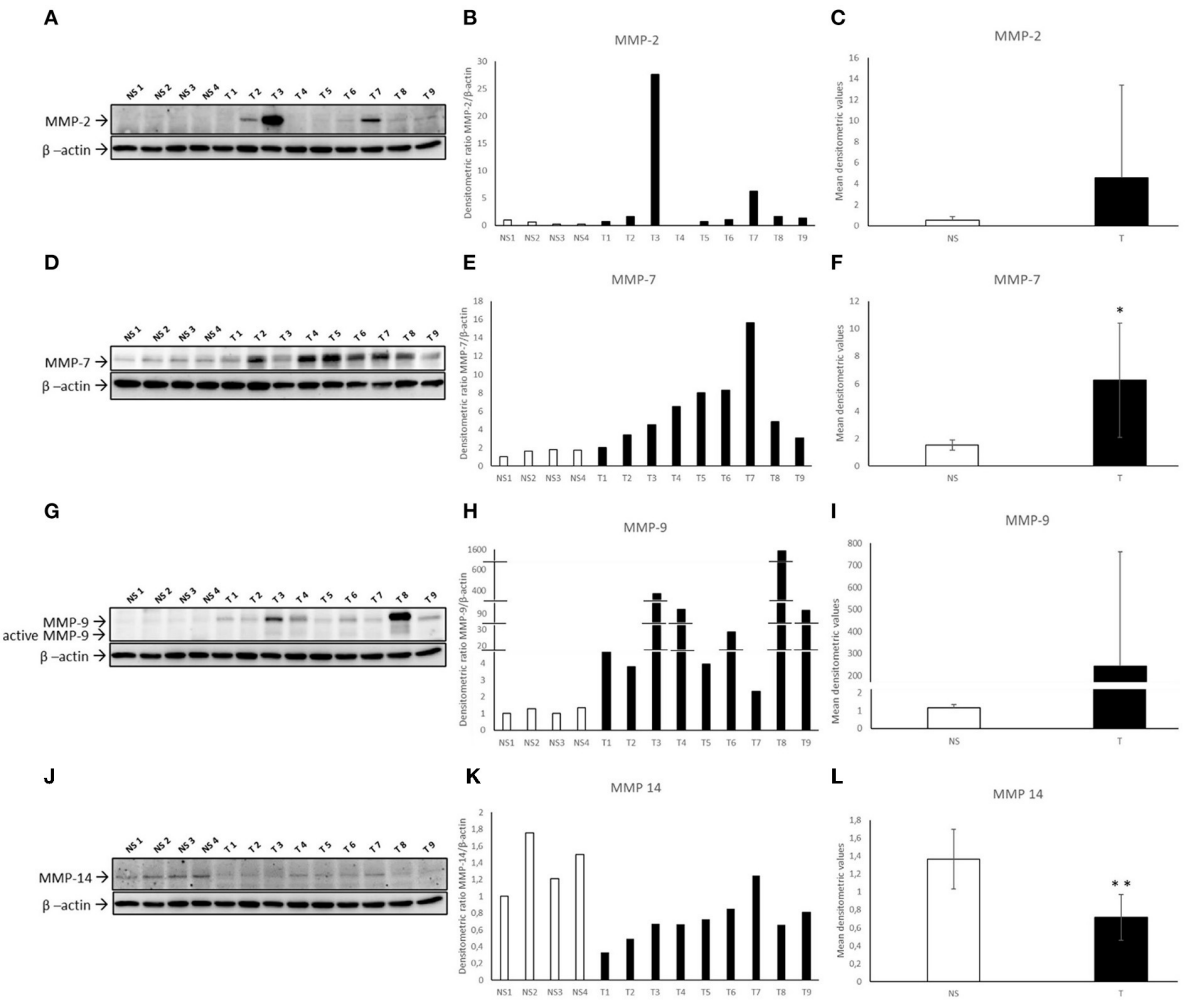
Western blotting analysis of MMP-2/-7/-9/-14 in bovine normal skin (NS) and fibropapillomas (T). Western blotting and densitometric measurements of MMP-2/-7/-9/-14 in NS1-NS4 and T1-T9 samples **(A–L)**. Representative gels showing an increased amount of MMP-2/-7/-9 **(A,D,G)** and decreased expression of MMP-14 **(J)** in tumor compared with normal skin samples are shown. The blots were stripped and reprobed with anti- β-actin antibody to confirm equal loading of proteins in each lane and allow normalization. **(B,E,H,K)** Individual densitometric values of MMP-2/-7/-9/-14 for each sample expressed as densitometric ratio with β-actin. **(C,F,I,L)** Mean densitometric values +/- standard deviations for MMP-2/-7/-9/-14 in NS vs T groups (*: statistically significant by *t*-test, *P* < 0.05; **: statistically significant by *t*-test, *P* < 0.01).

Nine bovine fibropapillomas (T1-T9) samples and four normal skin samples (NS1-NS4) were subjected to Western blotting analysis. The anti-MMP-2, anti-MMP-7, anti-MMP-9, anti-MMP-14, anti-TIMP-1 and anti-TIMP-2 antibodies yielded a band of expected molecular weight, confirming the specificity of IHC staining. MMP-2 appeared over-expressed in 8 out of 9 tumor samples (89 %), with particularly high levels in sample T3, when compared to normal skin samples, where its expression was very low (Figure 8A). Densitometric analysis of individual samples (Figure 8B) as well as mean tumor vs. normal skin values (Figure 8C) confirmed the results, although no statistically significant difference between samples was recorded, mostly due to high variability in the fold of upregulation among fibropapillomas.

Further, an over-expression of MMP-7 was detected in all tumor samples (Figure 8D) as confirmed also by individual (Figure 8E) and mean (Figure 8F) densitometric values of tumor vs. normal skin samples, and the difference between the two groups was statistically significant (*t*-test; *P* < 0.05).

Similarly, all fibropapillomas showed an increased expression of MMP-9 pro-form of 92 kDa when compared to normal skin samples (Figure 8G). Data were confirmed by densitometric analysis of individual samples (Figure 8H) as well as mean tumor vs. normal skin values (Figure 8I), although the difference was not statistically significant, probably for the reason stated above for MMP-2. Next, an additional band of ~82 kDa, likely corresponding to MMP-9 active form was observable in tumor samples, while it was very faint to undetectable in normal skin specimens.

When MMP-14 was analyzed, its expression was readily detectable in all normal skin samples, whilst in 9/9 tumor samples (100%), a lower expression was evident and confirmed by densitometric analysis (Figures 8J,K). Mean densitometric values revealed a statistically significant difference (*t*-test; ^**^P < 0.01) (Figure 8L).

**Figure 9:**
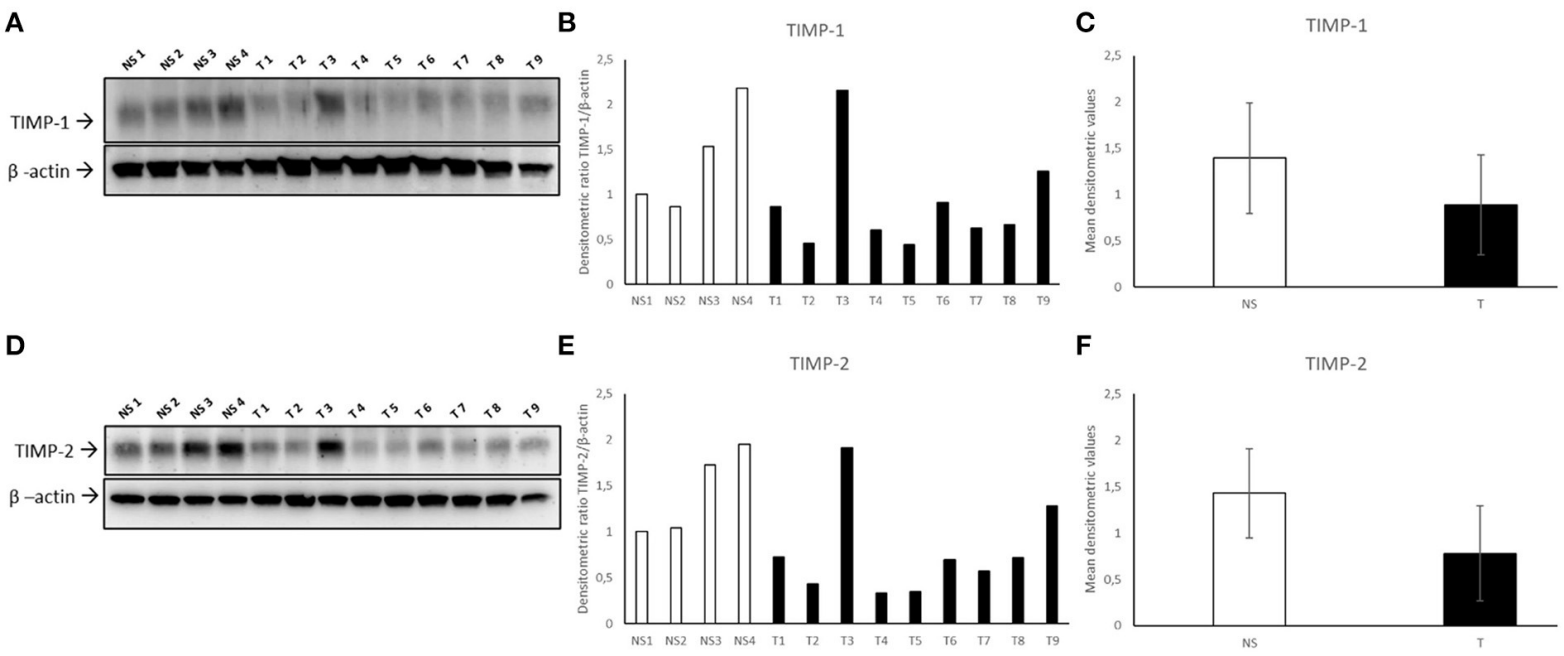
Western blotting analysis of TIMP-1/-2 in bovine normal skin (NS) and fibropapillomas (T). Western blotting and densitometric measurements of TIMP-1/-2 in NS1-NS4 and T1-T9 samples **(A–F)**. Representative gels showing a decreased amount of TIMP-1/-2 in tumor compared with normal skin samples are shown **(A,D)**. The blots were stripped and reprobed with anti- β-actin antibody to confirm comparable loading of proteins in each lane and allow normalization. **(B,E)** Individual densitometric values of TIMP-1/-2 for each sample expressed as densitometric ratio with β-actin. **(C,F)** Mean densitometric values +/- standard deviations for TIMP-1/-2 in NS vs. T groups.

TIMP-1 appeared down-regulated in most of fibropapillomas, when compared to normal skin samples (Figure 9A). Furthermore, densitometric analysis of individual samples (Figure 9B) as well as mean tumor vs. normal skin values (Figure 9C) confirmed the results, although the difference between samples was not statistically significant.

WB for TIMP-2 (Figure 9D) followed by densitometric analysis revealed very similar results (Figures 9E,F).

### Zymography

**Figure 10:**
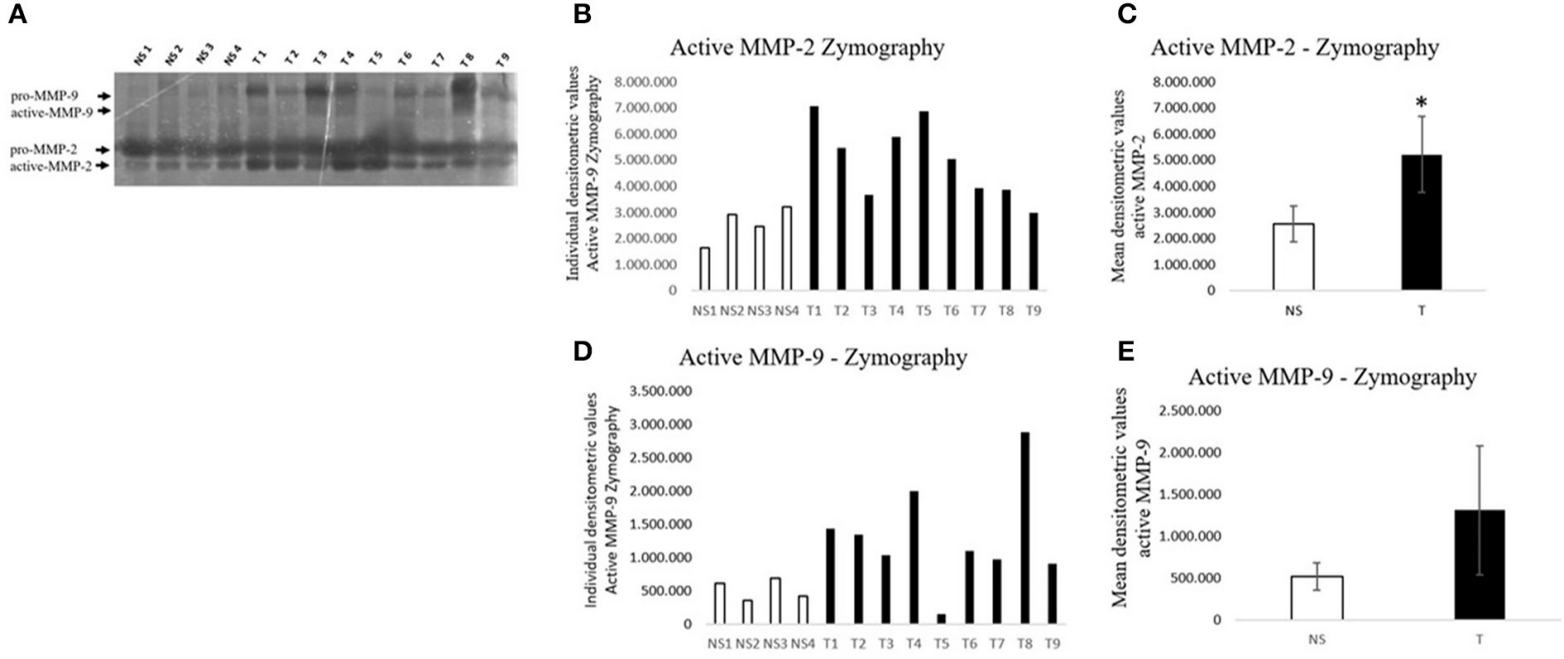
Zymographic assay of gelatinases activity in bovine fibropapillomas (T) and normal skin (N) samples **(A–E)**. **(A)** In all fibropapillomas, MMP-2 is present in both proform (72 kDa) and active form (62 kDa); in fibropapillomas the active form of MMP-9 (82 kDa) is present at higher levels. **(B,D)** Individual densitometric values of active MMP-2/-9 for each sample. **(E)** Mean densitometric values +/- standard deviations for active MMP-2/-9 in NS vs. T groups (*: statistically significant by *t*-test, *P* < 0.05).

In addition to protein expression levels, enzymatic activity of MMP-2 and MMP-9 was analyzed in normal skin and fibropapilloma samples using gelatin zymography. Pro- and active forms of MMP-2 were recorded with gelatin zymogram electrophoresis (Figure 10A). However, fibropapillomas showed an upregulation of the 62 kDa active MMP-2 in comparison with normal skin samples as confirmed by individual densitometric analysis, while the mean densitometric values revealed a statistically significant difference (Figures 10B,C) (*t*-test; ^*^P < 0.05). Impressively, when analyzing MMP-9 activity, its activated form of 82 kDa was readily detectable in all fibropapilloma, whilst it was present at low to undetectable levels in the normal skin samples, in agreement with western blotting results, denoting a strong activity during tumor development. Moreover, the individual and mean densitometric analysis revealed similar results as mentioned above, although no statistically significant difference was recorded (Figures 10D,E) (*t*-test; *P* < 0.05).

## Discussions

Bovine cutaneous fibropapillomas are common cutaneous tumors induced by BPV types−1/-2, and associated with lesions composed of mixed epithelial and mesenchymal tissue proliferation (30). Healthy bovids usually recover from fibropapillomas and if the animals are unable to overcome the infection due to various environmental or immunological factors, the tumor persistence may be seen (31). Indeed, tumor growth depends critically on the neoplastic proliferation and the ability of the tumor cells to produce different proteolytic enzymes and extracellular matrix proteins, such MMPs (32). Interestingly, MMPs and TIMPs have been investigated in equine sarcoids in terms of ECM remodeling and it was suggested that an aberrant expression of MMP genes along with altered transcriptional activities of MMPs and TIMPs genes can lead to imbalance between collagen synthesis and degradation processes and, consequently contributing to local invasion and impairment of extracellular matrix remodeling (15). The present study is the first to document the expression of matrix metalloproteinases (MMP-2, MMP-7, MMP-9, MMP-14) and their tissue inhibitors (TIMP-1 and TIMP-2) in bovine cutaneous fibropapillomas associated with BPV-2 infection.

Gelatinase A, secreted as pro-MMP-2 (72 kDa) and activated into the functional form (62 kDa), is expressed in a variety of normal and transformed cells (18). Our immunohistochemical and biochemical results showed that MMP-2 was expressed in fibropapillomas and moreover, pro- and active forms were recorded. Our data is consistent with previous results reported by Martano et al. (15), where MMP-2 was expressed in higher amount in equine sarcoids compared to normal skin (15). Moreover, a similar result is reported by Yuan et al. (33), demonstrating in equine sarcoids an over-expression of MMP-2, thus contributing to invasiveness of sarcoid fibroblasts (33). In human counterpart, active MMP-2 may serve as a predictive marker of metastasis and furthermore, the high activity of MMP-2 correlates with the invasiveness of oral SCC (34). Therefore, we speculate that MMP-2 may possess an important contribution during BPV induced fibropapilloma development.

Further, MMP-9, first termed 92-kDa type IV collagenase or gelatinase plays a major role in the degradation of ECM both in physiological and pathological processes (35). In bovine fibropapillomas, our results show for the first time the overexpression of pro- and active forms of MMP-9, which may possess an important proteolytic activity directed to different ECM proteins; by immunohistochemistry, MMP-9 has been detected both in epithelial and fibroblast cells, which may indicate a possible role in cell proliferation and detachment from basement membrane through an intense digestion of ECM proteins. In addition, a similar pattern of MMP-9 expression was described in bovine ocular squamous cell carcinomas, a tumor type attributed to different factors such as papillomavirus and herpesviruses, where an increased MMP-9 expression compared to the control group was demonstrated (36). In a recent *in vivo* study, Altamura et al. (37), showed that in feline oral squamous cell carcinoma cell lines, MMP-2 and−9 are expressed at gene and protein level, which in turn may suggest a contribution of feline oral squamous cell carcinoma cells to invasiveness, in agreement with studies on human oral squamous cell carcinomas, where MMPs expression may play a relevant role in determining the invasive phenotype of tumor cells (38).

Interestingly, we observed in tumor samples higher levels of MMP-2 and MMP-9, along with lower levels of MMP-14, their main activator. Recent studies have suggested that HPV-16 oncoproteins may promote cervical cancer invasiveness by upregulating two specific MMPs: MMP-2 and MMP-14 (39). It has been well accepted that MMP-14 is one of the major proteinases degrading the ECM during invasion. Therefore, in our study it is surprising that a decreased MMP-14 expression was detected, results which are in contrast with those reported by Martano et al. (15), where in BPV positive equine sarcoid the MMP-14 expression seems increased (15). However, MMP-14 was recently shown to act as an interstitial collagenase, and some recent studies reported this collagenase to be of major importance since deletion or suppression of MMP-14 in fibroblasts and tumor cells was associated with a loss of collagenolytic and invasive capacity *in vitro* and *in vivo* (40).

As with other MMPs, MMP-7 has been identified in a wide range of tumors. De Vicente et al. (41) studied MMP-7 in human oral squamous cell carcinoma (SCC) and found its expression in cancer cells, but not in normal oral epithelial cells (41). Moreover, MMP-7 was reported to be a malignant biomarker in ovarian cancer (42). Accordingly, we observed that MMP-7 was overexpressed in tumor samples, compared to normal skin. This is the first time when MMP-7 expression is reported in bovine BPV induced cutaneous tumors and our findings suggest that MMP-7 may play an important role in the development and progression of fibropapilloma.

In neoplastic tissues, the regulatory balance between MMPs and TIMPs is disturbed in favor of proteolysis (43). Interestingly, both tissue inhibitors of metalloproteinases (TIMP-1/-2) analyzed in this study were expressed in low quantities comparing to normal skin samples. In our study, a specific positivity for TIMP-1 was seen in tumor samples, which is in agreement with positivity reported in human oral squamous carcinomas (44–46). The balance between MMP-9 and TIMP-1 is thought to play a critical role in controlling extracellular-matrix turnover and the inhibition of tumor invasion and metastasis (47), therefore the decreased expression of TIMP-1 reported in our study may influence the tumor development and progression.

In the literature, much less and controversial data regarding TIMP-2 expression in papillomavirus induced tumors are available (15, 48). The present results clearly confirm the detection of TIMP-2 expression in normal skin samples, with a decreased expression in fibropapillomas. Our results are consistent with those reported by Sheu et al. (49) who detected only low levels of TIMP-2 in HPV induced cervical cancer, but contradictory with the results reported in equine sarcoid, where a TIMP-2 expression was evidently increased (15). This different expression level may depend on the proliferation stage, as was suggested in human counterpart, where TIMP-2 was shown to retain its normal expression pattern until late in disease progression, when 60% of cervical intra-epithelial neoplasia still expressed TIMP-2 approximately at the level of normal epithelium (48).

## Conclusion

This is the first study describing MMPs and TIMPs in bovine cutaneous fibropapillomas and our results suggest that their unbalanced expression in presence of BPV-2 may play a significant role in tumor development. A further analysis of supplementary MMPs and TIMPs could bring new important insights into the papillomavirus induced tumors.

## Data availability statement

The original contributions presented in the study are included in the article/supplementary material, further inquiries can be directed to the corresponding author.

## Ethics statement

The animal study was reviewed and approved by Ethics and Deontology Committee, Iasi University of Life Sciences Ion Ionescu de la Brad, Iaşi, Romania.

## Author contributions

FDB performed IHC. FDB and GA performed WB and densitometric analysis. AC performed zymography. OT, MD, OH, and SP performed IHC. FDB, GA, MM, and GB drafted the manuscript. All authors contributed to the article and approved the submitted version.

## Funding

This work was supported by a grant from the Ministry of Research, Innovation and Digitalization, CNCS/CCCDI_UEFISCDI, Project Number PN-III-P1-1.1-PD-2019-0040, 50/2020, within PNDI III.

## Conflict of interest

The authors declare that the research was conducted in the absence of any commercial or financial relationships that could be construed as a potential conflict of interest.

## Publisher's note

All claims expressed in this article are solely those of the authors and do not necessarily represent those of their affiliated organizations, or those of the publisher, the editors and the reviewers. Any product that may be evaluated in this article, or claim that may be made by its manufacturer, is not guaranteed or endorsed by the publisher.
